# Peer relationships buffer the negative association of online education with education satisfaction and subsequently with study engagement among undergraduate medical students

**DOI:** 10.1186/s12909-022-03337-3

**Published:** 2022-04-13

**Authors:** R. O. Wissing, F. Hilverda, R. A. Scheepers, A. P. Nieboer, M. Vollmann

**Affiliations:** Department of Socio-Medical Sciences, Erasmus School of Health Policy & Management, Erasmus University Rotterdam, Rotterdam, The Netherlands

**Keywords:** Peer relationships, Study context, Education satisfaction, Study engagement, Online education

## Abstract

**Background:**

Due to the COVID-19 pandemic, undergraduate medical students had to follow high amounts of online education. This does not match their preferences and might negatively affect their education satisfaction and study engagement. As low levels of education satisfaction and study engagement are risk factors for burnout and dropout, resources that mitigate these possible negative consequences of forced online education need to be identified. Therefore, the current study investigated 1) the associations of the amount of online education with education satisfaction and study engagement, and 2) whether quantitative (i.e., network size) and qualitative (i.e., perceived support) aspects of peer relationships can buffer the expected negative associations.

**Methods:**

In a cross-sectional study, 372 undergraduate medical students from all eight Dutch medical schools (79.8% female; mean age: 20.4 years) completed an online survey assessing the relevant variables. Data were analysed using correlation and moderated mediation analyses.

**Results:**

The amount of online education was significantly negatively related to education satisfaction and study engagement. Additionally, higher amounts of online education were indirectly associated with lower levels of study engagement through lower levels of education satisfaction. More importantly, both quantitative and qualitative aspects of peer relationships significantly buffered this negative indirect association. Specifically, among medical students with a large peer network or high levels of perceived peer support, the amount of online education was no longer significantly negatively related to education satisfaction and subsequently to study engagement.

**Conclusions:**

The current study underlines the importance of peer relationships in the educational context, since our findings indicate that both the peer network size and the perceived peer support protect medical students’ education satisfaction and study engagement when confronted with study demands, such as forced online education during the COVID-19 pandemic. These findings may be translated into educational efforts that stimulate collaborative learning and the formation of formal peer networks.

## Background

Due to the COVID-19 pandemic, undergraduate medical students were forced to complete high amounts of their education online as during the lockdowns, the educational offer mainly consisted of recorded lectures and knowledge clips, online discussion boards, and lectures and tutorials via collaboration and video conferencing platforms such as Zoom, Teams, and Kaltura [1]. However, empirical findings indicate that medical students prefer conventional face-to-face and blended education over online education [2–5]. Thus, online education during the COVID-19 pandemic did not match the preferences of medical students and can therefore be considered as an external stressor or study demand. Such a distressing mismatch can potentially lead to lower levels of education satisfaction and study engagement [6–11], which are important risk factors for burnout and dropout [12, 13]. These negative outcomes should be prevented as they are associated with severe consequences for students (e.g., economic loss, reduced self-confidence) [14–16] and the healthcare system (e.g., future lack of physicians, lower quality of care) [17–19]. Therefore, factors that can mitigate or even eliminate the potential negative impact of online education on education satisfaction and study engagement need to be identified and used to make recommendations about the design of online education.

### Impact of online education on education satisfaction and study engagement

Several theoretical frameworks, e.g., the theory of educational productivity [20] and the distance education student satisfaction model [21], underline the importance of the study context and whether it matches the preferences of students as determinants of study-related outcomes [20–24]. Similarly, previous empirical findings have demonstrated that a mismatch between the study context and personal preferences lowers education satisfaction [2, 6–8]. Additionally, empirical studies based on the job demands-resources model applied to the study context have shown that study demands are negatively associated with study satisfaction and study engagement [12, 25–27].

Little is known about the direction of the relationship between education satisfaction and study engagement. Previous occupational research has identified job satisfaction as an antecedent of work engagement [28–30]. Moreover, it has been theoretically argued and empirically supported that job satisfaction is a mediator between job characteristics and work-related outcomes [28, 31]. These findings suggest that study demands might be related to lower study engagement through lower education satisfaction. This mediating process has not yet been investigated in the context of study stress resulting from a mismatch between study preferences and the actual study context.

### Peer relationships as a buffer of the negative effects of forced online education on education satisfaction and study engagement

Various theoretical approaches, e.g., the stress-buffering hypothesis [32] and the job demands-resources model [33], consider quantitative and qualitative aspects of social relationships as resources that can protect individuals from diverse negative effects of external stressors. Empirical studies among (medical) students and healthcare professionals have confirmed that social relationships can mitigate or even eliminate the effects of demands or stressors on outcomes such as burnout, well-being, motivation, exhaustion, and academic or job performance [34–41]. By applying these findings to the context of online education during the COVID-19 pandemic, social relationships with fellow students might protect medical students from the negative consequences of large amounts of forced online education, such as reduced education satisfaction and study engagement.

### The present study

**Fig. 1:**
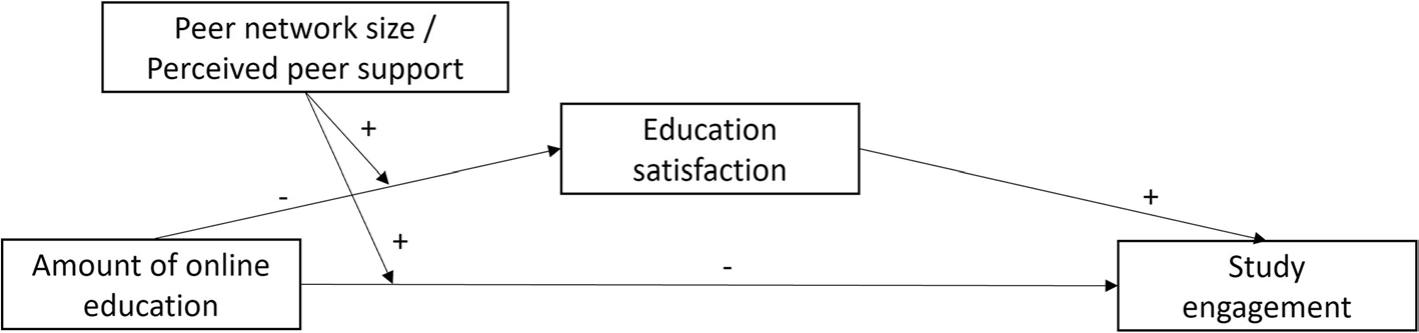
Expected mediated relationships among the amount of online education, education satisfaction, and study engagement as well as the moderating effects of peer network size and perceived peer support

The first aim of this study was to examine the relationships among the amount of online education, education satisfaction, and study engagement in undergraduate medical students. It was expected that a higher amount of online education is related to lower levels of education satisfaction and study engagement. It was also hypothesized that education satisfaction and study engagement are positively associated and that education satisfaction serves as a mediator between the amount of online education and study engagement. The second aim was to investigate whether quantitative (i.e., network size) and qualitative (i.e., perceived support) aspects of peer relationships moderate the expected indirect association between the amount of online education and study engagement through education satisfaction. It was expected that both peer network size and perceived peer support buffer this negative indirect association. More specifically, it was hypothesized that the negative indirect effect of the amount of online education on education satisfaction and subsequently on study engagement becomes weaker or disappears with an increasing peer network size and higher levels of perceived peer support. Figure 1 graphically represents the hypothesized moderated mediation relationships.

## Methods

### Procedure

This cross-sectional study was conducted during the lockdown in April 2021 via an online survey programmed in Qualtrics. Undergraduate medical students from all eight registered Dutch medical schools were eligible to participate. The participants were recruited via posts on social media (e.g., Facebook groups of medical students, Instagram, LinkedIn) and emails sent to members of various medical student associations. After opening the link to the questionnaire, participants had to complete an informed consent that highlighted their voluntary participation and anonymity. As compensation, participants who fully completed the questionnaire could take part in a raffle for gift vouchers. The study protocol was approved by the Medical Ethics Review Committee of the Erasmus Medical Center (#2020–0815).

### Measures

The survey was administered in Dutch. The means and standard deviations for all measures are shown in Table 1.

**Table 1:** Means, standard deviations, and bivariate correlations of study variables

	1	2	3	4	5
1. Amount of online education^a^					
2. Education satisfaction^b^	−.25^***^				
3. Study engagement^c^	−.12^*^	.27^***^			
4. Peer network size	−.09	.14^**^	.06		
5. Perceived peer support^c^	−.13^*^	.25^***^	.22^***^	.26^***^	
*M*	90.45	5.6	2.88	7.05	3.96
*SD*	6.51	1.81	0.98	5.79	1.21

*Note.* ^a^ Scale range: 0–100. ^b^ Scale range: 0–10. ^c^ Scale range: 0–6

^*^p < .05, ^**^p < .01, ^***^p < .001

#### Online education

The amount of online education was measured by a single item, i.e., “Since the beginning of the current academic year, what percentage of all your study activities has taken place online?”. Responses were given as a percentage ranging from 0 to 100%.

#### Education satisfaction

Education satisfaction was measured by a single item, i.e., “All things considered, how satisfied are you with your education since the beginning of the current academic year as a whole?”. Responses were given on an 11-point scale ranging from 0 (extremely dissatisfied) to 10 (extremely satisfied). Previous research supports the use of a single item to measure satisfaction [42, 43].

#### Study engagement

Study engagement since the beginning of the academic year was measured by the ultra-short Utrecht Work Engagement Scale (UWES-3), consisting of three items [44, 45]. The three items were adapted for application to the students’ academic lives. Responses were given on a 7-point scale from 0 (never) to 6 (always). Items were averaged, with higher scores indicating greater study engagement. In the current study, Cronbach’s alpha was .61.

#### Peer network size

The peer network size was measured by a single item, i.e., “With how many fellow students did you have frequent study-related and/or personal contact in the last month? This may have been face-to-face, but also digital.”. Responses were registered in an open-ended numerical format. The use of such a single-item measure for network size has been validated in previous research [46].

#### Perceived peer support

Perceived peer support since the beginning of the academic year was measured by using a three-item measure, focusing on emotional, informational, and practical support [47]. The responses were given on a 7-point scale from 0 (never) to 6 (always). Items were averaged, with higher scores indicating greater perceived peer support. Previous research supports the reliability and validity of this scale to measure perceived peer support [48]. In the current study, Cronbach’s alpha was .85.

### Data analysis

Statistical analyses were performed with IBM SPSS Statistics version 27 [49] and PROCESS macro for SPSS version 3.5.3 [50]. First, bivariate associations between the study variables were analysed using Pearson correlation analysis. Subsequently, two moderated mediation analyses (one for each moderator) with the amount of online education as the independent variable, education satisfaction as the mediator, study engagement as the dependent variable, and peer network size or perceived peer support as the moderator of the relation between the amount of online education and education satisfaction and study engagement were calculated using Hayes’ model 8 (Fig. 1).

The moderated mediation analysis contained the following subanalyses. First, a multiple regression analysis was conducted with education satisfaction as the dependent variable to estimate the effects of the amount of online education, the moderator (peer network size or perceived peer support) as well as the interaction effect between online education and the moderator. In the case of a significant interaction effect, the conditional effects of the amount of online education on education satisfaction were calculated for a low, medium, and high level (i.e., −1*SD*, mean, and + 1*SD*) of the moderator. Second, a multiple regression analysis was conducted with study engagement as the dependent variable to estimate the effects of the amount of online education, education satisfaction, the moderator (peer network size or perceived peer support) as well as the interaction effect between online education and the moderator. In the case of a significant interaction effect, the conditional effects of the amount of online education on study engagement were calculated for a low, medium, and high level (i.e., −1*SD*, mean, and + 1*SD*) of the moderator. Third, the index of moderated mediation was determined using bootstrapping with 5000 bootstrap samples, which indicated whether the indirect effect of the amount of online education on study engagement through education satisfaction varies depending on the level of the moderator. In the case of a significant index of moderated mediation, the conditional indirect effects of the amount of online education on study engagement through education satisfaction were estimated for a low, medium, and high level (i.e., −1*SD*, mean, and + 1*SD*) of the moderator, also using bootstrapping with 5000 bootstrap samples. Moreover, pairwise comparisons of the indirect effects were calculated to examine which of the indirect effects differ significantly from each other. Unstandardized coefficients are reported.

## Results

### Participants

A total of 637 medical students started the survey. After screening out respondents who did not meet the inclusion criteria (*n* = 68) and excluding respondents who did not finish the questionnaire (*n* = 153) or failed one of the various reliability checks, such as an inadequate processing time or implausible answers (*n* = 44), 372 participants were included in the analysis.

The sample consisted of 297 women (79.8%), 73 men (19.6%), and 2 individuals who identified as nonbinary (0.5%), with a mean age of 20.4 years (*SD* = 1.81, range: 17–31 years). Students from all eight registered Dutch medical schools participated and were in their first (29.6%), second (37.6%), or third (32.8%) undergraduate year. Most of the participants lived in student housing (41.7%) or with their parents/family (34.9%), followed by living alone (10.2%) and with friends (7.0%). The rest lived with their partner (4.0%), rented from a landlady (0.3%), or in another form (1.9%).

### Bivariate associations between study variables

The results of the correlation analyses are presented in Table 1. As expected, the amount of online education was significantly negatively associated with education satisfaction and study engagement. In addition, education satisfaction and study engagement were significantly positively related.

### Moderated mediation analyses

The multiple regression analyses with education satisfaction as the dependent variable (Table 2, columns 2 and 3) revealed significant interaction effects between the amount of online education and both moderators, indicating that the effect of the amount of online education on education satisfaction depends on the peer network size and the level of perceived peer support. More specifically, the conditional effects demonstrated that the amount of online education was significantly negatively related to education satisfaction at a low and medium level of peer network size or perceived peer support, but this relationship was no longer significant at a high level of peer network size or perceived peer support (Table 3, columns 2 and 3).

**Table 2:** Results of the multiple regression analyses as part of the moderated mediation analyses with education satisfaction and study engagement as dependent variables

Predictors	Dependent variable: Education satisfaction		Dependent variable: Study engagement	
	Moderator: Peer network size	Moderator: Perceived peer support	Moderator: Peer network size	Moderator: Perceived peer support
	***b***	***b***	***b***	***b***
Amount of online education	−0.10^***^	−0.18^**^	−0.02	0.02
Moderator	−0.43	−2.43^*^	−0.17	0.68
Amount of online education*Moderator	0.01^*^	0.03^**^	0.00	−0.01
Education satisfaction			0.14^***^	0.13^***^
	*R*^2^ = .09, *F*(3, 368) = 11.52^***^	*R*^2^ = .13, *F*(3, 368) = 18.01^***^	*R*^2^ = .08, *F*(4, 367) = 8.22^***^	*R*^2^ = .10, *F*(4, 367) = 10.39^***^

*Note.* ^*^p < .05. ^**^p < .01. ^***^p < .001

**Table 3:** Conditional effects of the amount of online education on education satisfaction and on study engagement via education satisfaction on the different levels of peer network size and perceived peer support

Level of moderator (peer network size / perceived peer support)	Conditional effects of amount of online education on education satisfaction		Conditional indirect effects of amount of online education on study engagement through education satisfaction	
	Moderator: Peer network size	Moderator: Perceived peer support	Moderator: Peer network size	Moderator: Perceived peer support
	***b***	***b***	***b*** (95% CI)	***b*** (95% CI)
–1*SD*: 1.26 / 2.75	−0.10^***^	−0.10^***^	−0.013 (−0.021, −0.007)	−0.013 (−0.020, −0.006)
*M*: 7.05 / 3.96	−0.07^***^	−0.06^***^	−0.009 (−0.013, −0.005)	−0.008 (−0.012, −0.004)
+1*SD*: 12.84 / 5.17	−0.04	−0.03	−0.005 (−0.010, 0.002)	−0.003 (−0.008, 0.001)

*Note.* Conditional indirect effects are significant at *p* < .05 when zero is not included in the 95% confidence interval (95% CI). ^***^p < .001

The multiple regression analyses with study engagement as the dependent variable (Table 2, columns 4 and 5) revealed nonsignificant interaction effects between the amount of online education and both moderators, indicating that the direct effect of the amount of online education on study engagement does not depend on peer relationships; this effect was not significant. However, a significant positive effect of education satisfaction on study engagement was found.

The index of moderated mediation was significant in both moderated mediation analyses with peer network size as the moderator, *b* = 0.0007, 95% *CI* (0.0001, 0.0017), and with perceived peer support as the moderator, *b* = 0.0038, 95% *CI* (0.0011, 0.0075). These significant indices indicate that the negative indirect effect of the amount of online education on study engagement through education satisfaction depends on the peer network size and the level of perceived peer support. Additionally, the pairwise comparisons between the conditional indirect effects were all significant. Overall, the negative indirect effect of the amount of online education on study engagement via education satisfaction became weaker with a larger peer network size or higher levels of perceived peer support (Table 3, columns 4 and 5). More specifically, the amount of online education had a significant negative effect on study engagement through lower education satisfaction at a low and medium level of peer network size or perceived peer support; however, this effect was no longer significant at a high level of peer network size or perceived peer support.

The results did not change significantly after controlling for age and gender (data not shown).

## Discussion

The present study found that online education was negatively associated with both education satisfaction and study engagement. These findings are in line with previous results indicating that forced online education has a negative effect on study-related outcomes, such as education satisfaction and study engagement [2, 6–10]. Moreover, the present findings extend previous research [28–31] by revealing mediating processes, i.e., the negative effect of online education on study engagement is fully mediated by education satisfaction. In agreement with several theoretical frameworks [20, 21] and empirical results [2, 6–8, 20–24], the present findings underline the importance of the study context and whether it matches the preferences of medical students, since a mismatch negatively affects their education satisfaction and subsequently their study engagement.

Most importantly, the present study identified peer relationships as a resource that can mitigate or even eliminate the negative effects of forced online education. As expected, both quantitative and qualitative aspects of peer relationships moderated the negative indirect effect of the amount of online education on study engagement through education satisfaction. More specifically, in medical students with large peer networks or with high levels of perceived peer support, online education was not associated with lower education satisfaction and subsequently lower study engagement. These results are in line with various theoretical frameworks [32, 33] and previous empirical studies [34–41], and emphasize the importance of peer relationships in the educational context.

### Practical implications

The results of the present study demonstrate that medical students enrolled in forced online education programs benefit from having a large peer network and from perceiving high levels of peer support. Consequently, medical schools should focus on facilitating opportunities for medical students to have more frequent and more meaningful interactions with their peers when they are forced to complete high amounts of their education online [51]. Implementing this physically without violating the governmental rules for social distancing during a lockdown is a challenge. Nevertheless, medical schools should stimulate social interactions between students via digital media in at least two ways. First, they should implement collaborative learning [24], e.g., use interactive education tools, create breakout rooms during online education, or set up more group assignments. Second, medical schools should also focus on setting up formal peer networks, for instance by implementing near-peer mentor groups [52], peer-led support programs [53, 54], peer support workshops [55], or virtual peer support group conferences [56]. Both approaches can stimulate social interactions between students, thus leading to higher satisfaction among students in online learning environments [24].

### Limitations

The present results must be considered in light of certain study limitations. First, due to the cross-sectional design, the direction of causality between education satisfaction and study engagement could not be determined [57]. Although the tested moderated mediation model is based on theoretical assumptions and empirical findings, it could not be excluded that the direction of the relationships between education satisfaction and study engagement might be reversed or reciprocal [58–60]. Second, education design characteristics (such as interactivity, collaboration, and synchronicity) were disregarded, despite their close relationship with education satisfaction and social relations [21, 24, 61, 62]. For example, education design characteristics can be moderating variables on the relationship between the amount of online education and education satisfaction [63]. Third, the present convenience sample limited the generalizability of the findings. Due to the self-selected sampling, the extent to which this sample is representative of the population of Dutch undergraduate medical students remains unclear. However, students from all eight registered Dutch medical schools did participate. Also, the sample mainly consists of female participants, but this is largely in line with the composition of medical students in the Netherlands [64]. Therefore, longitudinal and experimental studies with representative samples that also include education design characteristics are warranted in the future.

## Conclusions

This work underlines the importance of the study context and whether it matches the preferences of medical students, since a mismatch has a negative impact on study-related outcomes. Specifically, the present findings indicate that an increase in the amount of online education can result in decreased study engagement through reduced education satisfaction, which are known risk factors for burnout and dropout. Most importantly, the current study shows the relevance of peer relationships in the educational context. Medical students with large peer networks or high levels of perceived peer support do not lose education satisfaction and study engagement when they are forced to complete a large percentage of their education online.

## Data Availability

The datasets analysed during the current study are available in the Erasmus University Rotterdam’s data repository: 10.25397/eur.18257165 [65].
